# Advances in Genetic Transformation of *Lotus corniculatus*: Methodological Determinants, Applications and Future Priorities

**DOI:** 10.3390/plants15162520

**Published:** 2026-08-20

**Authors:** Chen Zhou, Jinghao Han, Shanhua Lyu, Haiyun Li, Yinglun Fan

**Affiliations:** College of Agriculture and Biology, Liaocheng University, Liaocheng 252000, China

**Keywords:** *Lotus corniculatus*, genetic transformation, *Agrobacterium*, bioreactor, stress resistance breeding, quality improvement

## Abstract

*Lotus corniculatus* is a superior leguminous forage with multiple values including forage, ecological, ornamental and medicinal uses. It is also an ideal material for plant bioreactors. As a core technical approach, genetic transformation overcomes the constraints of traditional breeding and facilitates the targeted improvement in stress resistance and agronomic traits in this species. This review summarizes the research progress of the *Agrobacterium*-mediated genetic transformation of *L. corniculatus*, focusing on key procedures such as explant selection, strain selection, infection and co-cultivation regimes, basal medium composition, phytohormone regulation, as well as bacteria elimination and transformant screening strategies. We further elaborate on the applications of this transformation system in enhancing tolerance to abiotic stresses (salt, drought and heat), regulating quality-related traits, and developing plant-based vaccine bioreactors. Additionally, this paper critically discusses the major bottlenecks and challenges constraining existing genetic transformation systems in *L. corniculatus*, and evaluates the prospects for establishing high-efficiency and genetically stable transformation platforms. This review aims to provide theoretical foundations and technical references for germplasm innovation, molecular breeding and comprehensive utilization of *L. corniculatus*.

## 1. Introduction

*Lotus corniculatus*, commonly known as bird’s-foot trefoil, is a perennial herbaceous species belonging to the genus *Lotus* within the *Fabaceae* family. It is widely distributed across temperate and subtropical regions worldwide [1], and represents an important plant resource with great value in agricultural production, ecological restoration and landscape application. It possesses a well-developed root system with dense lateral roots and exhibits strong stress tolerance and environmental adaptability, enabling it to serve as a pioneer species for marginal land improvement, soil and water conservation, and the restoration of degraded ecosystems [2]. Meanwhile, *L. corniculatus* exhibits a prolonged flowering period, conferring notable ornamental value. It demonstrates high tolerance to trampling and shade, low maintenance requirements, and strong resistance to diseases and insect pests. Therefore, it is suitable as a groundcover in roadside greening, urban parks, and suburban green spaces, indicating its broad potential for application in sustainable landscape management [3]. As a premium leguminous forage, *L. corniculatus* possesses well-balanced nutritional composition in its stems and leaves, characterized by low crude fiber content and adequate soluble carbohydrate reserves. Additionally, it is rich in condensed tannins, which not only enhance palatability and feeding safety but also effectively reduce the incidence of bloat in ruminant livestock. Owing to its strong regenerative capacity and excellent grazing tolerance, *L. corniculatus* is regarded as a highly promising staple forage species for temperate regions, with considerable potential to partially replace white clover (*Trifolium repens*) and alfalfa (*Medicago sativa*) in certain production systems [4].

*L. corniculatus* is characterized by prolific flowering, stable heritable traits, and high cellular totipotency, enabling efficient plant regeneration through embryogenic and adventitious shoot differentiation pathways. Based on its well-established genetic transformation system, *L. corniculatus* has been widely used to explore the regulatory mechanism of flavonoid metabolism, symbiotic nitrogen fixation, and stress tolerance, as well as in forage quality improvement. Meanwhile, it acts as an ideal recipient material for plant-based bioreactors, which can be used to produce veterinary vaccines against pathogenic microorganisms including foot-and-mouth disease virus and avian influenza virus, as well as a variety of recombinant therapeutic proteins [5,6,7]. Additionally, secondary metabolites derived from *L. corniculatus* exhibit documented antioxidant, DNA-protective, and anti-tumor activities, thereby signifying considerable developmental potential within the pharmaceutical and health-related sectors [8].

Although regarding genomic advancement, *L. corniculatus*, *Medicago truncatula*, and *L. japonicus* are all established legume models; *L. corniculatus* is distinguished by its allotetraploid genome and self-incompatibility. Consequently, compared to these diploid counterparts, *L. corniculatus* lags behind in terms of genomic resources, gene editing tools, and transformation efficiency.

Notwithstanding these favorable attributes, the practical production and large-scale application of *L. corniculatus* still face considerable bottlenecks. Under natural conditions, the species is also susceptible to abiotic stresses including drought, low temperature, salinity and alkalinity, which hampers field establishment and severely restricts its large-scale utilization in marginal lands and stress-prone environments. Moreover, conventional breeding is constrained by long breeding cycles, low efficiency, and the slow pyramiding of superior agronomic traits, rendering rapid, targeted, and efficient germplasm improvement in *L. corniculatus* inherently difficult. Within the broader landscape of modern crop breeding, genetic transformation plays a complementary and indispensable role alongside other advanced technologies. While genomic selection accelerates the prediction and selection of complex polygenic traits using genome-wide marker data, and genome editing enables precise, targeted modification of endogenous loci, genetic transformation serve as the foundational delivery platform for genome editing components and remains the sole viable approach for introducing novel traits from heterologous sources. With advances in molecular biology and genetic engineering, genetic transformation has emerged as an effective strategy to overcome the inherent constraints of conventional breeding, and is now a cornerstone technology for germplasm innovation, enhancement of abiotic and biotic stress tolerance, and improvement in agronomic and nutritional quality in *L. corniculatus*.

So far, the *Agrobacterium*-mediated stable genetic transformation system of *L. corniculatus* has been successfully established and progressively refined, which lays an important technical foundation for the introduction of exogenous genes, gene function verification and molecular breeding. Although substantial progress has been made in this area, the genetic transformation of *L. corniculatus* continues to be hampered by low transformation efficiency and pronounced genotype dependence, which collectively restrict its broader application. Although molecular biology advances in *L. corniculatus* have previously been reviewed in 2019, genetic transformation was discussed only as a brief subsection [9].

This review focuses specifically on genetic transformation systems, offering a critical and standardized evaluation of relevant experimental protocols. Recent innovations are discussed, including *DsRed2*-assisted transgenic identification and vermiculite-based rooting [10], *RUBY*-based hairy root screening [11], folate biofortification [12], heat shock protein engineering [13], and initial attempts at genome editing. It must be noted that the optimized transformation parameters detailed in specific studies are genotype-, explant-, and strain-specific unless broadly validated.

To systematically summarize advances in the genetic transformation of *L. corniculatus*, a targeted literature search was conducted using Web of Science, Scopus, PubMed, and the China National Knowledge Infrastructure (CNKI) databases, covering publications from 1980 to June 2026. Search terms included combinations of “*Lotus corniculatus*” with “genetic transformation,” “*Agrobacterium tumefaciens*,” “*Agrobacterium rhizogenes*,” “tissue culture,” “hairy root,” “transgenic,” “bioreactor,” “gene editing,” and “CRISPR/Cas9.” The inclusion criteria were: (1) peer-reviewed original research or review articles directly reporting genetic transformation of *L. corniculatus*; (2) studies on related legume species providing methodological insights transferable to *L. corniculatus*; and (3) reports containing quantifiable transformation parameters (explant type, basal medium, hormone regimen, selection system, and transformation efficiency). The reference lists of retrieved articles were manually screened to identify additional relevant studies.

Based on this systematic literature search, this paper reviews the main methods and key influencing factors of genetic transformation in *L. corniculatus*, summarizes research progress in stress-resistant genetic engineering and plant-based bioreactor application, identifies critical technical bottlenecks, and delineates future research directions and development prospects. This review aims to provide systematic theoretical guidance and technical reference for establishing an efficient genetic transformation system, innovating germplasm resources, and promoting the industrial application of *L. corniculatus*.

## 2. Genetic Transformation System of *L. corniculatus*

**Figure 1:**
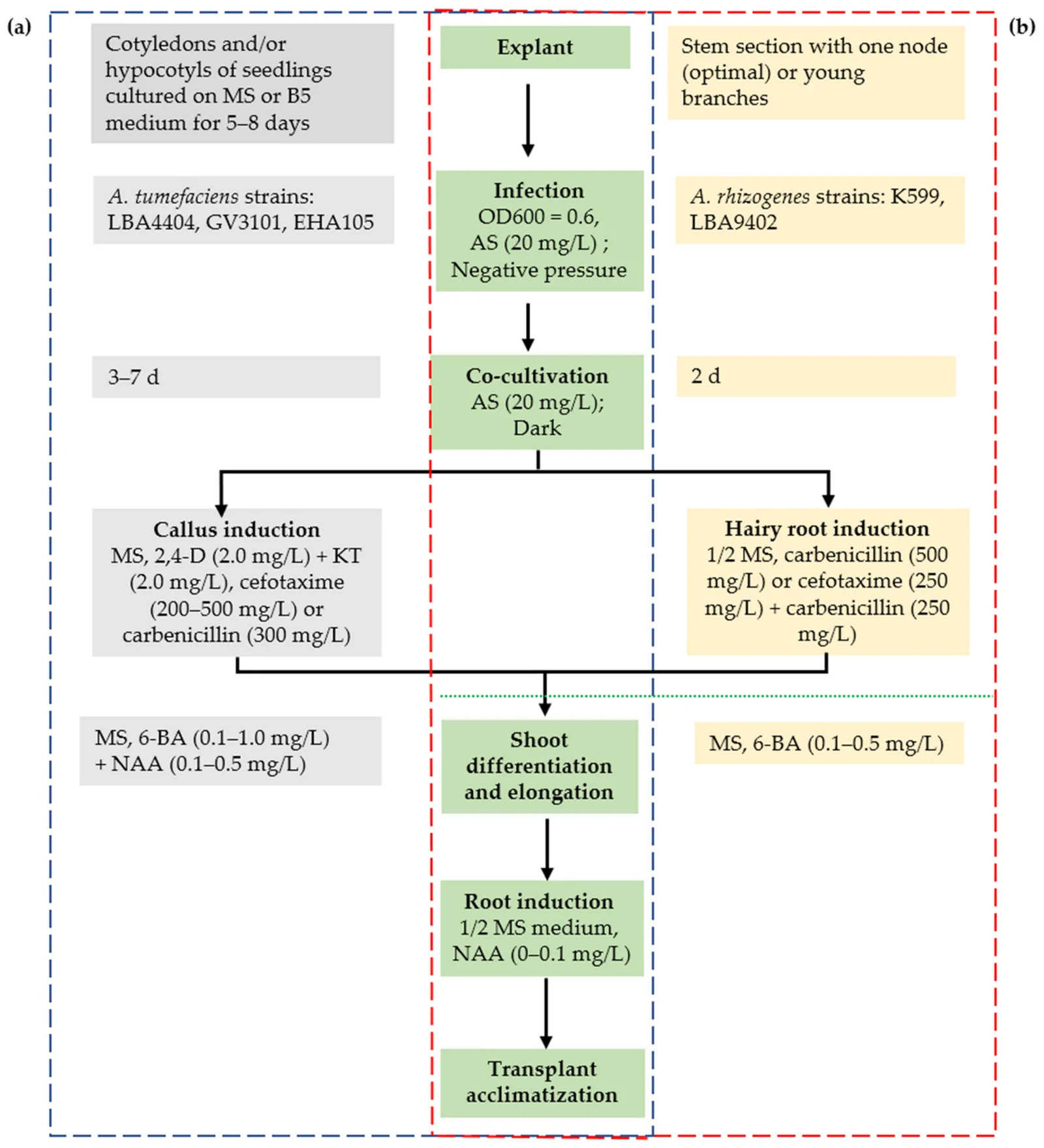
Schematic workflow of two *Agrobacterium*-mediated genetic transformation systems for *L. corniculatus*. (**a**) The blue dashed box illustrates the *A. tumefaciens*-mediated stable transformation system, which uses cotyledon and hypocotyl explants to produce heritable transgenic lines. (**b**) The red dashed box demonstrates *A. rhizogenes*-mediated hairy root transformation (above the green dashed line) and plant regeneration, supporting rapid hairy root induction for functional analysis and subsequent whole-plant regeneration (below the green dashed line). Black solid arrows indicate sequential experimental steps.

The genetic transformation techniques currently employed for *L. corniculatus* primarily include *Agrobacterium*-mediated methods and laser microbeam-mediated transformation. Among them, the *Agrobacterium*-mediated method has become the most mature and widely adopted core technology for *L. corniculatus* (Figure 1), owing to its operational simplicity, low exogenous gene copy number (usually 1–2 copies), and stable transgenerational inheritance [10,14]. *Agrobacterium* strains employed for genetic transformation of *L. corniculatus* are primarily classified into *A. tumefaciens* and *A. rhizogenes*. *A. tumefaciens*-mediated transformation is widely utilized to generate stable transgenic lines with heritable transgene expression (Figure 1a), yet it suffers from relatively low transformation efficiency. In contrast, *A. rhizogenes*-mediated transformation is commonly used to induce hairy root formation (referred to as hairy root transformation), which serves as a platform for transient gene expression analysis and functional studies of root-specific traits (Figure 1b), transgenic hairy roots can also be directly regenerated into complete transgenic plants [15,16]. The *Agrobacterium*-mediated transformation efficiency is co-regulated by multiple factors, including the bacterial strain used, explant type, infection and co-cultivation conditions, as well as selection and regeneration systems used.

### 2.1. Explant

The selection and treatment of explants constitute pivotal determinants for the successful genetic transformation of *L. corniculatus*, exerting a direct influence on both transformation efficiency and the quality of regenerated plantlets. Cotyledons, hypocotyls, leaves, stem segments and roots are conventional explant materials for this species, which should be chosen to fit the transformation system and experimental goals.

In *A. tumefaciens*-mediated genetic transformation of *L. corniculatus*, cotyledons, hypocotyls and leaves are generally used as explants. Cotyledons are favored as the primary explant source due to their high differentiation capacity, ease of isolation from surface-sterilized seedlings, and broad compatibility with diverse *A. tumefaciens* strains. Consequently, they have been extensively adopted in foundational and routine transformation protocols [1,3,17,18,19] (Table 1). Recent investigations have demonstrated that the transformation efficiency (the ratio of the number of independent transgenic lines confirmed by molecular analysis to the total number of explants infected, expressed as a percentage) of hypocotyls in the *L. corniculatus* cultivar ‘Leo’ slightly exceeds that of cotyledons [10]. Therefore, both cotyledons and hypocotyls can be concurrently used as explants for genetic transformation of *L. corniculatus*. The physiological status of explants directly influences transformation efficiency; when using cotyledons and hypocotyls as explants, the appropriate seedling age is 5–8 days [6,9,20], while seedlings aged 10–14 days are also acceptable [21]. Super-growing roots (SR), a rapid root culture system established in *L*. *corniculatus*, support perpetual root propagation, direct somatic embryogenesis and large-scale plant regeneration without exogenous plant growth regulators. This unique SR system expands explant options: leaves from SR-regenerated seedlings are adopted as explants in an indirect transformation method to generate transgenic superroots, which are subsequently regenerated into whole transgenic plants [16].

*A. rhizogenes*-mediated transformation typically uses stem segments as explants. For rapid induction of hairy roots, the optimal explant for *Agrobacterium*-mediated transformation is a stem section with one node, achieving a transformation frequency (the ratio of explants producing confirmed transformants to the total number of explants infected, expressed as a percentage) of 74.64%, significantly higher than that of roots (14.49%), internodes, and leaves [15]. Furthermore, the utility of stems as explants has been documented in three other studies [5,22,23]. A one-step cutting method was adopted using *L. corniculatus* ‘Leo’ as the experimental material to compare the transformation frequency of young branch and seedling explants [11]. The results demonstrated that composite plants derived from young branches exhibited a significantly higher positive hairy root transformation frequency (81.1%) than those derived from seedlings (62.2%); moreover, composite plants regenerated from young branches were stronger and taller than the plants obtained from the seedlings.

### 2.2. Strain Selection

The virulence and host compatibility of *Agrobacterium* strains are critical factors affecting the transformation efficiency of *L. corniculatus.* Currently, *Agrobacterium*-mediated transformation of this species mainly utilizes two types of strains: *A. tumefaciens* and *A. rhizogenes*. Different strains differ markedly in transformation characteristics, optimal reaction conditions and applicable scenarios; thus, strain selection should be rationally matched to specific experimental purposes (Table 1).

Among *A. tumefaciens* strains widely adopted for *L. corniculatus* transformation, LBA4404, GV3101 and EHA105 are the most prevalent [6,12,18,20,21,24,25,26,27]. LBA4404 represents an early strain established for this legume’s transformation system [21]. It possesses moderate virulence and steady growth traits, delivering excellent experimental repeatability, and has therefore become a foundational strain for routine transformation research. By contrast, EHA105 yields significantly higher transformation efficiency than LBA4404 and GV3101 [28]. In practical experimentation, EHA105 is prioritized for assays pursuing maximal transformation efficiency, while LBA4404 remains a reliable choice for routine transformation studies due to its steady growth and reproducible performance.

**Table 1:** Comparison of *Agrobacterium*-mediated transformation protocols for *L. corniculatus*.

No.	Cultivar	Explant/Age/ Number	Strain/Vector	OD_600_/Inf.	CC./(d)	AS (mg/L)	Selection Marker/Regime/(mg/L) or Reporter	Medium	PGRs (Diff., mg/L)	Metric (Definition) and Value (%)	Molecular Confirmation Method	Result	Ref.
1	‘Leo’	Cotyledons/5–10 d/200	C58C1/pGV3850:neo1103	—/ 10 min	2	—	*nptII* /Kan/10)	MS	6-BA/0.1	—	DNA dot blot	WP	[29]
2	‘Leo’	Cotyledons/5–8 d/310	AGL1 /pBin438	—/ 30 min	3	—	*nptII*/Kan/25–100	MS	6-BA/0.1	TF (explants with transformants/total explants infected) 9.7	PCR, Southern blot	WP	[30]
3	‘Viking’	Cotyledons and hypocotyls/10–14 d/200/	LBA4404 /pBI121	—/ 30 min	7	100	*nptII* /Kan/100	B5	6-BA/0.5	—	GUS, PCR	WP	[21]
4	—	Cotyledon/7d/—	LBA4404 /pCAMBIA3301	—/ 30 min	1	—	*Bar* /Basta/2	MS	6-BA/0.1	—	PCR	WP	[31]
5	‘Leo’	Cotyledons/5–8 d/—	EHA105, LBA4404, GV3101 /pRG229	0.5/ 30 min	3	20	*nptII*/Kan/50–100	MS	6-BA/0.1	TE (mol-confirmed independent lines/total explants infected) 12.5% (EHA105); 7.7% (LBA4404); 4.5% (GV3101)	PCR, PCR-Southern	WP	[28]
6	—	Hypocotyls/6–8 d/—	LBA4404 /VP60-pBI121	0.6/ 20 min	3	—	*nptII* /Kan/50	MS	6-BA/1.0 + NAA/0.1	—	PCR	WP	[24]
7	—	Cotyledons/7 d/—	EHA105 /pBHB	0.5/ —	—	—	*nptII* /Kan 50	MS	6-BA/0.1	—	PCR, Southern blot	WP	[27]
8	‘Mirabel’	Cotyledon with petiole/—	GV3101 /pCAMBIA1302	0.5/ 20 min	3	—	*hptII*/Hyg/15	B5	6-BA/0.5	—	PCR, RT-PCR	WP	[19]
9	‘Leo’	Cotyledon with petiole/—	EHA105 /pCAMBIA1302	0.5/ 20 min	3	—	*Bar* /Basta/—	B5	6-BA/0.5	—	PCR, RT-PCR, RT-qPCR	WP	[12]
10	‘Mirabel’	Cotyledon with petiole/—	GV3101 /pAVP1	0.5/ 20 min	3	—	*nptII* /Kan/50	B5	6-BA/0.5	—	PCR	WP	[32]
11	‘Leo’	Cotyledons/8 d/200	LBA4404 /pCAMBIA1302-PMI	0.6/ 20 min	3	—	*PMI*/mannose 20 g/L + sucrose 20 g/L	MS	6-BA/0.1	TE (PCR-positive independent lines/total explants infected) 16.2%	PCR, Southern blot, RT-PCR	WP	[1]
12	‘Leo’	Cotyledons/8 d/—	LBA4404 /pBI-MARs-HA	—/ 20 min	3		*nptII* /Kan/100	MS	6-BA/0.1	TF (Southern-positive lines/total shoots induced) 58.8%	PCR, Southern blot, RT-PCR, Western blot	WP	[6]
13	—	Hypocotyls/7–10 d/100	—	—	—	—	—	MS	6-BA/1.0 + NAA/0.5	—	—	WP	[33]
14	‘Leo’	Leaves/—/—	—	—	—	—	—	MS	KT/0.3	—	—	WP	[34]
15	‘Leo’	Cotyledons/—/—	GV3101 /pH7WG2D	—/20 min	3	—	*hptII* /Hyg/—	MS	6-BA/0.1	—	PCR, qPCR	WP	[3]
16	‘Leo’	Cotyledons/7 d/—	LBA4404 /pCAMBIA2301	—/20 min	3	—	*nptII* /Kan/50	MS	6-BA/0.1	—	PCR	WP	[17]
17	‘Leo’	Cotyledons and hypocotyls/7 d/—	LBA4404 /pR35BTR1	0.6/ 20 min	3	—	*DsRed2*/visual	MS	6-BA/1.0 + NAA/0.2	TE (confirmed independent lines/total explants infected) 16.67 (cotyledons); 18.89 (hypocotyls)	PCR, DsRed2	WP	[10]
18	‘Viking’	Leaves from SR-regenerated seedlings/—/919	LBA4404 /pBI121	—/ 30 min	7	100	*nptII* /Kan/100	MS	6-BA/0.5	—	GUS staining, PCR	WP from SR	[16]
19	‘Franco’,	Stems, petioles and leaves/8–10 weeks/—	LBA9402 /pRi1855 + AGS125	—	—	—	*hptII*/Hyg/20	MS	NAA/1.0 + 6-BA/1.0	—	Southern blot	WP from HR	[23]
20	‘Leo’	Stems/—/—	LBA9402	—	—	—	*nptII* /Kan/—	1/2 B5	—	HRIF (explants producing hairy roots/total explants infected) 60	PCR, qRT-PCR	WP from HR	[5]
21	‘Leo’	Stems/—/—	1855 /pBI121.1	—	—	—	*nptII* /Kan/50	1/2 B5	—	—	Southern blot, RT-PCR,	WP from HR	[22]
22	‘Leo’	Cotyledons/5–8d/198	Y-112	—/ 30 min	3	—	*nptII* /Kan/25	MS	6-BA/0.1	HRIF (explants producing hairy roots/total explants infected) 45.5	Dot blot, Southern blot	WP from HR	[35]
23	‘Viking’	Stem section with one node/—/—	K599 /pGFPGUSPlus	0.6/ 30 min	2	—	*hptII*/Hyg/4	MS	6-BA/0.5	HRIF (explants producing hairy roots/total explants infected) 91.5	GUS staining, PCR, Southern blot, Western blot	WP from HR	[15]
24	‘Leo’	Young branch; Seedling/—/30	K599 /p35RUBY	—	—	—	*RUBY*/visual	—	—	THRTF (explants producing composite plants/total explants infected) 81.1 (young branch); 62.2 (seedling)	RUBY, PCR	Composite plants	[11]

Entries 1–18 represent *A. tumefaciens*-mediated transformation, while 19–24 (shaded gray) represent *A. rhizogenes*-mediated transformation. No., number; Inf., Infection time; CC., Co-cultivation; AS, Acetosyringone; PGRs, Plant growth regulators; Diff., Differentiation; Ref., References. *nptII*, Neomycin phosphotransferase II; *Bar*, Phosphinothricin acetyltransferase; *hptII*, Hygromycin phosphotransferase II; *PMI*, Phosphomannose isomerase; *DsRed2*, Red fluorescent protein reporter; Kan, Kanamycin; Hyg, Hygromycin; Basta, Glufosinate-ammonium; MS, Murashige and Skoog medium; B5, Gamborg B5 medium; 6-BA, 6-benzylaminopurine; NAA, 1-Naphthaleneacetic acid; KT, Kinetin; TE, Transformation efficiency; TF, Transformation frequency; HRIF, Hairy root induction frequency; THRTF, Transgenic hairy root transformation frequencies; WP, Whole plant; HRC, Hairy root culture; WP from SR, SR-derived explant → callus → super root → shoot → rooting; WP from HR, Explant → hairy root induction → shoot organogenesis → rooting; Composite plants, Non-transgenic plant with transgenic hairy roots (root-only transformation); “—“indicates the corresponding data were not reported in the original literature.

Importantly, a higher initial transformation frequency does not necessarily equate to greater stability of transgene inheritance. To date, systematic multigenerational comparison of inheritance patterns specific to different strains has not been reported in *L. corniculatus*.

Apart from *A. tumefaciens*, *A. rhizogenes* serves as another transformation vector for *L. corniculatus*. Early studies confirmed that the A4M70GUS strain could infect root segment explants of the *L. corniculatus* cultivar ‘Bokor’ and generate transgenic regenerant plants [4], demonstrating the feasibility of the *A. rhizogenes*-mediated transformation system. Additionally, the LBA9402 strain has been successfully used to mediate transformation of hairy roots in *L. corniculatus* [5,26,36]. Notably, strain K599 is extensively utilized to generate transgenic hairy roots [11,15]. Coupled with the easy-to-operate, high-efficiency SR regeneration system, this strain supports a fast, high-yield protocol for recovering transgenic plants from ‘Viking’ superroots [15]. Overall, A4M70GUS, LBA9402 and K599 are three classic *A. rhizogenes* strains applied to transform *L. corniculatus*, among which K599 exhibits broader applicability when matched with the high-efficiency SR regeneration system.

### 2.3. Infection and Co-Cultivation

Infection and co-cultivation are critical steps for the transfer and integration of T-DNA from *Agrobacterium* strains (including *A. tumefaciens* and *A. rhizogenes*) into *L. corniculatus* cells. The concentration of bacterial solution, co-culture time and the selection of inducing substances directly affect the transformation efficiency and the survival rate of explants. The concentration of *Agrobacterium* solution during infection directly determines the number of *Agrobacterium* cells in contact with target cells, which in turn affects the infection efficiency and the survival rate of explant cells. An *OD*_600_ of 0.6 is the optimal concentration applicable to both *A. rhizogenes* K599-mediated transformation of stem sections with one node from Superroot-derived plants and *A. tumefaciens*-mediated transformation of cotyledons and hypocotyls. This *OD*_600_ recommendation is supported by several independent studies employing different *Agrobacterium* species [1,10,15], though systematic dose–response curves across multiple genotypes have not been reported. However, this value may vary depending on the tested genotype, explant type and bacterial strain. When the bacterial suspension is too dilute (*OD*_600_ < 0.4), insufficient *Agrobacterium* cell density leads to reduced T-DNA delivery efficiency, significantly impairing transformation efficiency. Conversely, excessively high concentrations (*OD*_600_ > 0.8) impose physiological stress on explants, leading to toxic reactions, manifested as intensified browning or even death of the explants. Therefore, the bacterial suspension concentration should be strictly maintained within an appropriate range. Surfactants can enhance the permeability of cell membrane and facilitate the adhesion of *Agrobacterium* to the wound of explants. It has been reported that Silwet L-77 can significantly increase the transformation frequency of *Brassica napus* [37]. This observation was made in *B. napus;* whether surfactants can be used to improve *Agrobacterium*-mediated infection efficiency in *L. corniculatus* remains to be verified.

Negative pressure-assisted infection is an effective physical method to improve the *Agrobacterium*-mediated transformation efficiency. Negative pressure treatment can cause slight trauma on the surface of explants, promote the release of plant phenolic signal substances, enhance the chemotaxis and adhesion of *Agrobacterium* to injured tissues, and then improve the efficiency of T-DNA transfer and integration. However, too high negative pressure intensity will cause irreversible damage to explants and inhibit callus differentiation and adventitious bud regeneration. Therefore, it is necessary to optimize the negative pressure parameters according to the type of plant materials. In *L. corniculatus*, using 5-day-old cotyledons as explants and treating them with −0.05 MPa negative pressure for 5 min increased the genetic transformation efficiency of the EHA105 strain from 13.8% to 19.0% [28]. Overall, appropriate negative pressure application can substantially enhance genetic transformation efficiency.

During the co-cultivation of *Agrobacterium* and plant explants, the addition of phenolic compounds to activate the expression of *virulence* (*Vir*) genes is a conventional strategy for improving genetic transformation efficiency. Among these phenolic inducers, acetosyringone (AS) exhibits the most pronounced inductive effect [38]. In the *A. tumefaciens* EHA105-mediated transformation system using cotyledons from *L. corniculatus* ‘Leo’ as explants, supplementation with 20 mg/L AS yielded the highest transformation efficiency. Efficiency declined progressively at concentrations exceeding 40 mg/L, and 100 mg/L AS caused severe browning and complete necrosis in explants during co-cultivation [28]. In another study, 100 mg/L AS was used for LBA4404-mediated transformation of *L. corniculatus* [16]. Besides AS, novel inducing agents have been explored in recent years. Treatment with 5 μmol/L chloroxynil, a synthetic organic compound used primarily as a herbicide, was shown to increase the transient transformation efficiency of *L. japonicus* to 60-fold that of the blank control and 6-fold that of the 100 μmol/L AS treatment group [39]. It is important to note that this result was obtained in *L. japonicus*, a related legume, and should be considered an extrapolation to *L. corniculatus* until directly verified. After all, the application of chloroxynil in the genetic transformation of *L. corniculatus* has not yet been reported.

Co-cultivation duration should be adjusted according to explant type and bacterial strain characteristics. For *A. tumefaciens*-mediated transformation of cotyledon and hypocotyl explants, a co-cultivation period ranging from 3 to 7 days has been widely adopted in multiple studies [10,21,28,30]. In contrast, the transformation system mediated by *A. rhizogenes* K599 with superroot-derived stem sections with one node as explants yields a peak transformation frequency of 91.5% with a 2-day co-cultivation period. Shortening the co-cultivation duration to 1 day or extending it to 3–4 days reduces the transformation frequency to less than 60% [15].

### 2.4. Basal Medium and Hormones

The precise regulation of medium components and plant growth regulators is the core factor that determines the efficiency of explant dedifferentiation, redifferentiation and *Agrobacterium*-mediated T-DNA integration. At present, MS and B5 as the basal medium in the genetic transformation of *L. corniculatus.* The frequently utilized plant growth regulators consist of auxins, including 2,4-dichlorophenoxyacetic acid (2,4-D) and α-naphthaleneacetic acid (NAA), as well as cytokinins represented by 6-benzylaminopurine (6-BA) and kinetin (KT). In different transformation stages such as callus induction, adventitious bud differentiation and rooting, the hormone ratio and concentration are different, and need to match the explant type and transformation system (Table 1).

Hormone-free MS or B5 medium is commonly employed at the seed germination stage to cultivate vigorous, aseptic seedlings [6,10], while MS medium is conventionally used as the basal medium during co-cultivation [40]. For callus induction, 2,4-D is mainly applied in combination with cytokinins. During adventitious shoot differentiation, cytokinins serve as the primary regulators supplemented with a low concentration of auxin. Among these combinations, 6-BA at 0.1–1.0 mg/L combined with NAA at 0.1–0.5 mg/L represents a classic formulation for adventitious shoot induction and is well-supported by multiple studies [10,24,33].

At the rooting stage, a low concentration of NAA (0.0–0.1 mg/L) is used to promote the normal development of root system. In the rooting and acclimation stage, cytokinins are completely eliminated, and only an extremely low concentration of auxin is retained or no growth regulators are added at all. In some protocols, half-strength MS (1/2 MS) medium is adopted to attenuate the growth dominance of aerial tissues and facilitate the initiation of adventitious root primordia.

In recent years, a novel rooting system consisting of liquid MS medium supplemented with NAA and vermiculite has been developed to replace traditional agar-solidified media. This system improves rhizosphere aeration, increases the rooting rate of transgenic plantlets, promotes their growth, and improves the survival rate of transplanting [10]. Although reported only in ‘Leo’, this method should be applicable to other cultivars as well.

### 2.5. Bactericidal Treatment and Screening System

Bactericidal treatment and positive screening are the key steps to determine the authenticity and survival rate of transgenic plants in *Agrobacterium*-mediated genetic transformation. *L. corniculatus* is relatively sensitive to exogenous antibiotics. Therefore, optimizing the type and concentration of bactericidal agents as well as screening intensity is of great significance for reducing explant browning and improving the acquisition rate of positive transgenic plantlets.

#### 2.5.1. Bactericidal System

Bactericidal treatment following *Agrobacterium* infection mainly relies on bactericidal antibiotics to suppress the growth of residual *Agrobacterium*, while avoiding phytotoxicity to plant cells. At present, cefotaxime and carbenicillin are the most commonly used bactericidal agents in *L*. *corniculatus* transformation, with common application concentrations ranging from 250 to 500 mg/L.

In *A. tumefaciens*-mediated transformation system, cefotaxime at concentrations ranging from 200 to 500 mg/L [1,10,20,28] or carbenicillin at 300 mg/L [32] is commonly used for bactericidal treatments post-co-cultivation. These dosages effectively restrain bacterial proliferation without exerting deleterious effects on de novo shoot organogenesis or subsequent plantlet regeneration.

For Superroot induction via *A. rhizogenes* K599, either 500 μg/mL carbenicillin alone or a combination of 250 mg/L cephalexin and 250 mg/L carbenicillin completely suppresses *A. rhizogenes* growth. Both regimens also markedly alleviate explant browning and elevate hairy root induction efficiency [15,41]. For K599-mediated hairy root transformation, no antibiotic treatment is required, and the infected explants are directly transplanted in sterile vermiculite wetted with sterile water [11].

Studies have demonstrated that an excessively high concentration of bactericidal agents delays explant regeneration, whereas an insufficient concentration easily leads to *Agrobacterium* regrowth. Accordingly, the dosage should be flexibly adjusted according to bacterial strain virulence and explant type.

#### 2.5.2. Screening Markers and Screening System

Genetic transformation of *L. corniculatus* predominantly relies on two selection strategies: antibiotic-based selection and biosafe positive selection. At present, antibiotic-based selection remains the most widely adopted method; however, biosafety-compatible positive selection systems are increasingly gaining traction as a promising alternative. In addition to selectable markers, reporter and visual-screening systems play a complementary role in verifying transgenic events.

Antibiotic-based selection systems predominantly utilize hygromycin, kanamycin, and glufosinate. Hygromycin B is a highly stable and efficacious selective agent for the genetic transformation of leguminous species [15,18,23,42], with a commonly used concentration of 15–20 mg/L [23,40]. A reduced concentration of 4 mg/L hygromycin B is sufficient to completely suppress the proliferation of non-transformed cells, while permitting the normal differentiation of transgenic cells [15]. Kanamycin is widely used for the screening of the *nptII* marker gene at a common concentration of 50–100 mg/L. Although this methodology necessitates a prolonged selection period, it exhibits broad adaptability to different genotypes [6,15,40]. When the *Bar* gene is used as the selective marker, glufosinate ammonium selection yields a high proportion of positive plants with stable inheritance in subsequent generations, and 2 mg/L is the most suitable selection concentration for *L. corniculatus* [31].

Although conventional antibiotic-based selection (e.g., hygromycin, kanamycin) is highly efficient, its application is subject to biosafety-related regulatory restrictions in certain countries and regions. This has driven the development of positive selection system without resistance markers, among which a mannose-dependent system mediated by the phosphomannose isomerase (*PMI*) gene has been successfully implemented. On medium supplemented with 20 g/L mannose and 20 g/L sucrose, non-transformed cells fail to metabolize mannose and undergo growth arrest, while transformed cells proceed through normal differentiation, yielding a transformation frequency of up to 16.2% [1]. It should be acknowledged that both antibiotic-resistance markers and PMI function as positive selection markers that allow transformed cells to survive or grow preferentially. They differ only in mechanism; antibiotic markers eliminate non-transformed cells, whereas PMI provides a metabolic advantage that favors transformed cell growth.

In parallel, reporter and visual-screening systems facilitate the detection and verification of transgenic events, but their mechanisms of action differ from those of selectable markers. β-glucuronidase (GUS) enables histochemical detection of both transient and stable transgene expression [18,20]; the red fluorescent protein DsRed2 allows real-time, non-invasive identification of transgenic callus and adventitious buds via red fluorescence [10]; and the RUBY reporter system enables visual discrimination of transgenic hairy roots through betalain pigment biosynthesis, without requiring exogenous substrates or specialized instruments [11]. These reporter systems function solely as complementary tools for confirming transformation success, distinct from selection agents that dictate survival or growth rates.

In summary, antibiotic selection remains the primary method for genetic transformation in *L. corniculatus*, with hygromycin-mediated selection being widely adopted and validated across multiple independent studies [15,18,39,40]. In contrast, the PMI system offers a safer alternative with superior biosafety; however, PMI-based positive selection has been reported in only a single study to date [1]. Additionally, visualization reporter genes such as *GUS*, *DsRed2*, and *RUBY* provide validation tools at different stages of the transformation process. Once transformants have been identified through selection, the next critical step is to induce rooting from regenerated shoots to obtain complete plantlets.

### 2.6. Rooting Induction and Transplanting Acclimatization

During the conventional root induction phase, culture media utilizing agar as the gelling agent frequently exhibit poor air permeability, which inevitably leads to a high probability of contamination of the regenerated buds, slow rooting, weak root systems and low survival rate after transplantation [43,44]. Utilizing vermiculite as an alternative supporting matrix to agar effectively optimizes the rhizosphere microenvironment. This substitution significantly enhances root proliferation, mitigates contamination risks, and ultimately improves both the post-transplantation survival and subsequent vegetative growth of transgenic plantlets [10]. Additionally, a rapid and simple 4-step transformation protocol has been developed, in which elongated shoots (~2 cm) are excised from the base of the buds and directly transferred to pots containing growth media, thereby significantly shortening the transformation cycle of *L. japonicus*, tobacco (*Nicotiana tabacum*) and soybean (*Glycine max*) [45]. Although this protocol has not yet been experimentally validated in *L. corniculatus*, its demonstrated adaptability across various species suggests that it may also be adapted for the genetic transformation of *L. corniculatus* to reduce the overall transformation timeline.

According to published literature, the most reproducible *A. tumefaciens*-mediated transformation protocol for *L. corniculatus* involves infecting cotyledons or hypocotyls from 5 to 8-day-old seedlings with LBA4404 or EHA105 (*OD*_600_ = 0.6). After a 3-day co-cultivation on MS medium with acetosyringone, shoots are induced on MS medium containing 0.1 mg/L 6-BA and rooted on MS or 1/2 MS medium. For *A. rhizogenes*-mediated hairy root transformation, infecting stems with K599 (*OD*_600_ = 0.6) and co-cultivating for 2 days offers the highest level of experimental reproducibility.

### 2.7. Genome Resources and Transformation in Other Lotus Species

Unlike well-characterized congeners, the allotetraploid *L. corniculatus* lacks a high-quality chromosome-scale nuclear genome. Available resources are restricted to organelle genomes (chloroplast: ~150,700 bp; mitochondrial: ~401,300 bp) and resequencing/GWAS data from 272 accessions that identified candidate genes for cyanogenic glycoside and proanthocyanidin biosynthesis [46,47,48]. Conversely, the diploid model *L. japonicus* offers complete chromosome-level assemblies, extensive multi-omics datasets, and advanced transformation platforms not found in *L. corniculatus* [49,50]. Its stable and hairy root transformation protocols utilize explants and *Agrobacterium* strains similar to those used for *L. corniculatus*. Additionally, established CRISPR/Cas9 gene-editing vectors and biosafe PMI selection systems routinely deployed in *L. japonicus* serve as important technical references [51]; meanwhile, fundamental transformation procedures have been reported for the forage species *L. tenuis* [52]. Due to the scarcity of genomic data for *L. corniculatus*, comparative mapping with *L. japonicus* is the principal strategy for orthologous gene discovery. Overall, the resources and tools across the *Lotus* genus create a robust framework for molecular breeding in *L. corniculatus*, helping to overcome the challenges of its polyploid genome.

## 3. Research on Application of Genetic Transformation of *L. corniculatus*

### 3.1. Abiotic Stress Resistance Genetic Engineering

Based on well-established genetic transformation systems, including *A. tumefaciens*-mediated and *A. rhizogenes*-mediated transformation, significant advances have been achieved in abiotic stress-resistant genetic engineering of *L. corniculatus*. These studies address diverse abiotic stresses such as salt, drought, and extreme temperature, thereby providing valuable genetic resources and feasible improvement strategies for the molecular breeding of forage legumes (Table 2). However, the biological stress resistance (disease or insect resistance) engineering remains largely unexplored in this species.

Salt stress is a major abiotic constraint limiting the geographical distribution and biomass accumulation of *L. corniculatus*. Genetic engineering of key regulatory genes governing ion homeostasis, osmotic adjustment, and transcriptional reprogramming has proven effective in enhancing salt tolerance in transgenic *L. corniculatus*. Heterologous expression of the soybean *GmNHX1* has been shown to attenuate Na^+^ accumulation, maintain ionic homeostasis, and significantly improve both the survival rate and root vigor of *L. corniculatus* under salt stress [20]. In addition, the endogenous AP2/ERF transcription factor gene *LcERF056*, a member of the B3-1 (IX) ERF subfamily, was significantly up-regulated in response to salinity, and its overexpression positively regulated salt stress tolerance in this species [3].

Drought tolerance of *L. corniculatus* can be independently improved by modulating osmotic regulation pathways. The overexpression of the trehalose-6-phosphate synthase gene (*TPS1*) enhanced cellular osmotic adjustment capacity, thus alleviating drought-induced damage and improving the drought resistance of transgenic plants [54].

Notably, several genes confer dual tolerance to salt and drought stress, providing great potential for improving the comprehensive stress adaptability of *L. corniculatus*. Heterologous expression of *AtAVP1* enhanced vacuolar ion compartmentalization, maintained stable leaf water status and photosynthetic efficiency, and thereby simultaneously improved salt and drought tolerance [32]. Similarly, the co-expression of *ZxNHX* and *ZxVP1-1*—derived from the xerophyte *Zygophyllum xanthoxylum*—achieved a synergistic enhancement of both drought and salt tolerance via vacuolar ion compartmentalization [19]. Additionally, co-expression of *PeDREB2a* and *KcERF* transcription factors enhanced salt and drought resistance in *L. corniculatus*, effectively broadening its environmental adaptation range [17].

In addition to osmotic and ionic stress tolerance, temperature tolerance of *L. corniculatus* can be improved through functional regulation of heat shock proteins (HSPs). Overexpression of *LcHsp70-1* enhanced the dual tolerance to both high and low temperatures via chaperone-mediated protein quality control, reinforcement of antioxidant defense systems, and regulation of stress-responsive transcription factors [13].

### 3.2. Forage Quality and Safety Improvement

In parallel with the development of stress-resilience engineering, researchers have also begun to focus on the targeted improvement in the forage quality, nutritional value and safety of *L. corniculatus*. Genetic transformation studies aimed at improving *L. corniculatus* quality center on feed safety, nutritional quality, and palatability, with a focus on three main directions: precise regulation of condensed tannins (proanthocyanidins), functional nutrient enhancement such as folate, and modulation of cyanogenic glycosides as anti-nutritional and safety-related traits.

Condensed tannins are pivotal secondary metabolites that fundamentally determine the forage quality of *L. corniculatus*. Optimal PA concentrations are instrumental in mitigating ruminant tympany (bloat), exerting anthelmintic activity against gastrointestinal parasites, and attenuating enteric methane emissions. Conversely, excessive accumulation compromises palatability and dietary protein degradability [7,10]. Recent advancements in genetic engineering have enabled the directional activation and precise modulation of PA biosynthesis. For instance, heterologous expression of the strawberry transcriptional repressor gene *FaMYB1* in *L. corniculatus* allows its protein to competitively bind to the MYB-bHLH-WD40 (MBW) complex, thereby significantly downregulating the expression of *DFR*, *ANS*, *ANR* and *LAR1*. This approach achieves heritable and specific reduction in leaf PA content without altering PA accumulation in floral tissues or other flavonoid metabolic pathways, providing an effective strategy for palatability improvement [5]. Furthermore, overexpression of the maize bHLH transcription factor gene *Sn* in *L. corniculatus* significantly increased the accumulation of condensed tannin polymers in leaves, while antisense suppression of dihydroflavonol reductase (DFR) specifically downregulated PA synthesis, identifying DFR as a key node in tannin biosynthesis [22] (Table 2).

Recently, *LcMYB5*, an R2R3-MYB transcription factor, was identified as a master regulator of PA biosynthesis in *L. corniculatus*. Overexpression of its two homologs (*LcMYB5a* and *LcMYB5b*) in *L. japonicus* hairy roots upregulated key structural genes (*CHS*, *F3H*, *LAR*, *ANR*, and *TT15*) and regulatory genes (*TT8* and *TTG1*) in the PA pathway, leading to a significant increase in flavonol content [58]. Despite being obtained in the related model species *L. japonicus*, these results suggest that *LcMYB5* can be bioengineered for PA production in the leaves of forage species including *L. corniculatus*.

Cyanogenic glucosides (CNglcs) are also secondary metabolites that affect forage safety, as they release hydrogen cyanide (HCN) upon enzymatic hydrolysis, potentially causing poisoning or even death in grazing livestock [59]. Consequently, high CNglc concentrations represent an anti-nutritional and safety concern that constrains the utilization of certain *L. corniculatus* germplasm. A global resequencing study of 272 *L. corniculatus* accessions identified multiple candidate genes and SNP loci associated with CNglc accumulation via genome-wide association analysis. A non-synonymous mutation in *LjMTR* reduced the expression of the CNglc biosynthesis gene, while *LjZCD* has been shown to positively regulate the CNglc synthesis gene *CYP79D3* [48]. These genomic resources provide candidate genes and molecular markers for breeding low-CNglc varieties through transformation-assisted functional validation and molecular breeding, although direct metabolic engineering of CNglc biosynthesis in *L. corniculatus* has yet to be reported.

Folate biofortification, aimed at enhancing the forage nutritional profile—particularly with respect to essential vitamins and amino acids—has achieved substantial progress. The co-expression of *MsGCHI* and *MsADCS* genes (derived from *Medicago sativa*) in *L. corniculatus* significantly upregulates the biosynthesis of key folate precursors, namely pteridines and *p-aminobenzoic* acid (*p-ABA*). This metabolic engineering strategy significantly elevates the accumulation of biologically active folate derivatives, including 5-methyltetrahydrofolate (5-M-THF) and 5-formyltetrahydrofolate (5-F-THF). Total folate levels in the leaves and stems of transgenic lines reached 2.1-fold and 1.7-fold increases relative to the wild-type (WT), respectively. Furthermore, this genetic modification concomitantly stimulates plant growth, achieving a synergistic enhancement of both nutritional density and biomass accumulation [12]. This integrated system offers a replicable solution for enhancing the functional nutrition of forage grasses.

### 3.3. Bioreactor

Beyond the improvement in stress adaptability and agronomic quality traits, the potential of *L. corniculatus* as a plant bioreactor has attracted increasing attention. Characterized by rapid regeneration capability and direct edibility, *L. corniculatus* has been explored as a leguminous forage platform applied for the production of animal vaccine antigens and recombinant pharmaceutical proteins. Expression or integration of a viral antigen gene in *L. corniculatus* represents an initial step toward plant-based bioreactor development. However, the path from transgene integration to an effective edible vaccine requires multiple validation stages: transgene integration, transcript detection, antigen accumulation, structural/functional antigen validation, oral immunogenicity, protective efficacy in target animals, and field-scale production. Most existing studies have progressed only to the first two stages. Via *Agrobacterium*-mediated transformation, structural protein-encoding genes *VP1* and *P12A-3C* of the Foot-and-Mouth Disease Virus (FMDV) have been successfully integrated into the genome of *L. corniculatus* and the target proteins expressed correctly, providing proof of transgene expression for FMDV vaccine development [26]. Similarly, the hemagglutinin (*HA*) gene of the H5N1 subtype avian influenza virus has been successfully expressed in the forage legume species *L. corniculatus* ‘Leo’, marking a preliminary step in the development of edible vaccines for poultry [6]. Furthermore, the successful integration and transformation of the VP60 capsid protein from Rabbit Hemorrhagic Disease Virus (RHDV) and the Hepatitis B Surface Antigen (*HBsAg*) has expanded the range of heterologous proteins produced by this species [24,27]. Notably, a site-specific integration vector carrying the *E2* major antigen gene of hog cholera virus has been constructed for chloroplast transformation of *L. corniculatus* [60]; however, chloroplast integration, antigen accumulation and immunogenicity have not yet been demonstrated. It is worth noting that the aforementioned studies have not demonstrated oral immunogenicity or protective efficacy in target animals; thus, further validation involving antigen accumulation, functional verification, and animal trials is crucial for developing *L. corniculatus* as an edible vaccine platform. Furthermore, the dual identity of *L. corniculatus* as transgenic forage and vaccine bioreactor leads to overlapping, inconsistent GMO and pharmaceutical regulatory standards, alongside strict environmental containment rules to prevent transgene flow in open cultivation.

### 3.4. Symbiotic Nitrogen Fixation

As a leguminous forage, *L. corniculatus* establishes symbiotic associations with rhizobia, facilitating biological nitrogen fixation [61]. Notably, *L. corniculatus* was the first legume species utilized to investigate nodule gene function through genetic transformation. A chimeric soybean leghaemoglobin gene was introduced into *L. corniculatus* via *Agrobacterium*-mediated transformation, confirming its nodule-specific expression [55]. A 1.9 kb fragment from 5′-upstream region of the *Sesbania rostrata* hemoglobin gene (*glb3*) was also introduced into *L. corniculatus* via *Agrobacterium*-mediated transformation [56]. Subsequently, the successful introduction of the soybean lectin gene *Le1* into *L. corniculatus* further validated the feasibility of this method and revealed the function of lectins in host-symbiont recognition [57]. Despite these reports, the application of transformation-based methods in improving the nodulation efficiency or nitrogen fixation capacity of *L. corniculatus* still needs to be explored. In the future, leveraging the established nodulation regulatory network in *L. japonicus* could enable targeted engineering of symbiotic traits, potentially enhancing nitrogen use efficiency in *L. corniculatus* forage systems and promoting sustainable development.

### 3.5. Other Applications

Genetic transformation of *L. corniculatus* also serves as a valuable tool for analyzing the regulatory mechanism of plant development. Overexpression of *LcERF1* in *L. corniculatus* and *Arabidopsis thaliana* increased endogenous auxin levels in shoot tips and roots, significantly promoted the formation, root number and elongation of adventitious roots, and provided molecular insights for optimizing clonal propagation in leguminous species [25].

In addition to developmental regulation, gene transformation is also utilized to confer agronomic traits beneficial for field management. Introduction of the *Bar* gene into *L. corniculatus* via *Agrobacterium*-mediated transformation resulted in transgenic plants with strong resistance to the herbicide glufosinate, and the *Bar* gene was confirmed by PCR in regenerated plants [31].

### 3.6. Considerations for the Application of Transgenic Technology

Several critical considerations for genetic transformation applications in *L. corniculatus*, such as transgene containment and gene flow, promoter specificity, inducible expression, and recent genomic resources for target discovery, have received limited attention to date and deserve dedicated discussion.

*L. corniculatus* is highly self-sterile and pollination is made mainly by insects [62]. This characteristic raises potential biosafety concerns regarding gene flow. Field trials using transgenic *L. corniculatus* carrying the *Escherichia coli* asparagine synthetase gene *asn*A as a tracer marker confirmed intraspecific transgene flow, but reproductive isolation mechanisms may restrict transgene spread to its close relatives *L. tenuis* and *L. pedunculatus* [63]. Prior to large-scale field trials of transgenic lines, further studies on additional containment measures is required, including artificial induced male sterility, temporal or spatial deployment strategies.

In *L. corniculatus*, most transformation work relies on the constitutive CaMV 35S promoter [5,10], which disrupts metabolism and triggers a trade-off between growth and defense. Tissue-specific (leaf, root) and stress-induced regulatory sequences enable precise spatiotemporal control of transgenic expression. Although the characterized promoters from *L. japonicus* and related species serve as valuable references, the exploration and validation of endogenous promoters in *L. corniculatus* remain limited.

The advancement of *L. corniculatus* genomics has promoted the discovery of target genes for transgenic research. Resequencing of 272 global *L. corniculatus* accessions has identified relevant loci associated with CNglcs accumulation and growth traits [48], providing support for candidate gene mining which is crucial for advancing molecular breeding of *L. corniculatus*.

## 4. Problems and Expectation

Although the genetic transformation system of *L. corniculatus* has been progressively refined and holds considerable promise for enhancing stress tolerance, improving forage quality, and enabling plant-based bioproduction, critical challenges persist—including technical bottlenecks, insufficient regulatory precision, and a disconnect between fundamental mechanistic research and industrial application. These constraints collectively impede the robust transition of *L. corniculatus* into a high-value, commercially viable forage species.

### 4.1. Core Existing Challenges

Low transformation efficiency has long restricted the large-scale application of molecular breeding and functional gene research in *L. corniculatus*, representing the most prominent technical bottleneck in its genetic transformation system. Despite years of methodological optimization, the transformation efficiency mediated by *A. tumefaciens* remains consistently below 20% [10]. Beyond these empirically optimized parameters, inherent biological constraints likely contribute to the low transformation efficiency in *L. corniculatus*. Genotype- and explant-dependent differences in regeneration, along with poor dedifferentiation capacity of mature tissues, are common obstacles in legume plants [38]. As a tetraploid species, *L. corniculatus* harbors extensive heterochromatin that restricts T-DNA integration and transgene expression, with genotype-dependent chromatin accessibility further modulating transformation efficiency. Moreover, plant immune responses to *Agrobacterium*, including oxidative burst and defense hormone signaling, suppress T-DNA delivery and integration. Genotype-dependent balance between phenolic-induced *Agrobacterium* virulence and host defense tolerance explains why certain varieties exhibit superior transformability [64]. Additionally, T-DNA integration mechanisms (NHEJ/MMEJ) and insertion patterns in *L. corniculatus* remain poorly characterized. Epigenetic silencing represents another barrier, as CaMV 35S-driven multicopy transgenes frequently undergo methylation and heritable silencing [65]. The impact of RNA-directed DNA methylation and post-transcriptional silencing on stable expression across genotypes remains unclear. Profiling chromatin states in regenerable tissues, characterizing defense signaling during co-cultivation, and mapping T-DNA integration patterns are essential to overcome existing transformation bottlenecks.

Tissue culture procedures are labor-intensive and suffer from poor experimental reproducibility. The full cycle from explant infection to field-ready transgenic plants in *L. corniculatus* typically lasts 4–6 months or even longer. Moreover, the efficiency of callus induction and adventitious shoot differentiation is highly influenced by genotype, hormone composition, light conditions, and medium formulation, resulting in significant variability.

Genotype dependence and ploidy complexity present major barriers to transforming and editing in *L. corniculatus*. Cultivated material is commonly tetraploid, while some transformation and editing work uses diploid lines. This distinction directly affects regeneration, allele editing, segregation and recovery of genetically fixed material. A relevant study followed independent *L. corniculatus* lines through the T2 and T3 generations and found line- and tissue-dependent expression and multiple segregating inserts in some lines [66]. These findings suggest that relying solely on initial reporter-positive frequency is inadequate for confirming stable transformation; instead, multigenerational inheritance studies and molecular verification of copy number are essential. Furthermore, for genome editing in tetraploids, the main challenge lies in editing all four alleles simultaneously rather than achieving standard diploid homozygosity, thereby making the recovery of fixed lines substantially more difficult.

Deficiencies persist in the rooting and acclimatization stages of micropropagation. Conventional agar-solidified rooting media suffer from poor gas exchange, frequently inducing root hypoxia, tissue browning, and secondary microbial contamination. Transgenic plantlets generated from such culture systems develop fragile root architectures, leading to severe wilting and elevated mortality rates upon transplantation due to drastic environmental changes. Although agar can be replaced with vermiculite to ameliorate rooting conditions [10], rooting induction still relies on aseptic culture, and seedling mortality during transplant acclimatization remains unavoidable.

Insufficient precision in transgene regulation constitutes another critical limitation. Most existing studies still rely predominantly on constitutive strong promoters represented by CaMV 35S. Sustained, high-level expression of exogenous genes throughout the entire growth period and across all tissues perturbs endogenous metabolic homeostasis, thereby eliciting growth–defense trade-offs manifested as vegetative growth retardation, yield penalties, and compromised stress adaptability [17]. By contrast, the exploitation and application of tissue-specific and stress-inducible regulatory elements are severely insufficient, which greatly constrains the biosafety and practical efficiency of targeted genetic modification. Tissue-specific promoters can address these limitations by directing transgene expression to physiologically relevant tissues to enhance trait efficacy, restricting transgene products to non-edible tissues to improve biosafety, and utilizing diverse endogenous promoters to mitigate the homology-dependent co-suppression and methylation-mediated silencing prevalent in multicopy 35S-driven lines [65].

Systems for polygenic synergistic regulation and targeted genome editing remain underdeveloped in *L. corniculatus*. Complex agronomic traits including stress resistance and quality improvement are generally modulated by intricate, multilayered regulatory networks involving numerous genes. Nevertheless, the efficiencies of multi-gene co-transformation and gene pyramiding in *L. corniculatus* remain exceedingly low, which hinders the synergistic improvement in multiple metabolic pathways. Currently, there has been no report of an efficient genome editing platform for *L. corniculatus*. Therefore, establishing an efficient CRISPR/Cas9 system is an urgent task for *L. corniculatus.* The more relevant limitation in tetraploid germplasm may be recovery of edits across multiple alleles rather than homozygosity in the ordinary diploid sense. However, advanced technologies such as high-fidelity Cas variants, base editing, and prime editing have been validated in other legumes but not yet in *L. corniculatus*.

The translation of laboratory-scale innovations into scalable industrial production remains limited. Despite the validated technical feasibility of producing edible vaccines and pharmaceutical proteins using *L. corniculatus*-based bioreactors, a huge gap separates laboratory achievements from practical pasture utilization. Exogenous proteins account for only 0.1–1% of total soluble protein, falling far below the threshold required for commercial viability. Furthermore, critical parameters of edible vaccines, such as antigenic stability and oral immunogenicity, have yet to be comprehensively validated through large-scale field trials, hindering their application in veterinary medicine.

### 4.2. Future Perspectives

To address the aforementioned bottlenecks, future research on genetic transfor-mation of *L. corniculatus* should prioritize four strategic directions—enhanced efficiency, spatiotemporal precision, data-driven optimization, and scalable industrial integration—to enable transformative advancement.

Modulating the expression of shoot regeneration-related genes is a promising but largely untested strategy for enhancing genetic transformation efficiency in *L. corniculatus*. Overexpression of developmental regulatory factors has successfully overcome transformation difficulties in various species. *TaLAX1* promoted callus regeneration in wheat, and its homologs enhance regeneration competence in maize and soybean [67]. Heterologous expression of *BnBBM* derived from *Brassica napus* efficiently resolved the recalcitrant transformation bottleneck in sweet pepper (*Capsicum annuum*), thereby markedly elevating its transformation efficiency [68]. Furthermore, the *Arabidopsis AtPLT5* facilitated transformation in *Brassica rapa* and sweet pepper. Similarly, *Arabidopsis WUS*, *PLT5*, and *WIND1* improved transformation in snapdragons and tomato [69]. These findings suggest that mining and overexpressing endogenous homologs of *LAX1*, *BBM*, *WUS*, *PLT5*, and *WIND1* in *L. corniculatus*, or introducing heterologous regeneration-regulatory genes, may improve the regeneration efficiency. However, there are several problems that must be considered: (1) all the evidence cited derives from heterologous species—none of these regulators has been tested in *L. corniculatus*; (2) the effects of developmental regulators are often genotype- and species-specific, so findings in the above plants may not be directly applicable to *L. corniculatus*; (3) constitutive overexpression of *BBM* and *WUS* may lead to various abnormalities, including ectopic embryogenesis and sterility, thus requiring inducible or tissue-specific expression systems. Therefore, whether these regulatory factors can significantly improve regeneration in this species is still a problem to be proved by experiments.

Optimizing *Agrobacterium*-mediated inoculation with physical assistance and simplifying the transformation process are alternative feasible strategies to improve transformation efficiency. The optimization of *Agrobacterium* infection parameters, combined with physical enhancement approaches including vacuum infiltration and ultrasound-assisted inoculation, can substantially improve transformation efficiency. When combined with a streamlined four-step transformation protocol that permits direct transplantation without in vitro rooting induction, these advances collectively can reduce the transformation period and markedly enhance post-transplantation seedling survival rates [45].

Overcoming genotype dependence represents a further critical priority for broadening the applicability of *L. corniculatus* transformation. Several emerging strategies present potential solutions to this challenge. First, exogenous application of the wound signaling peptide REGENERATION FACTOR1 (REF1), recently identified as a conserved regulator in plant regeneration, has enhanced transformation efficiency by 4- to 5-fold in recalcitrant crops such as soybean, wheat, and maize [70]. Second, a fast and genotype-independent in *planta Agrobacterium*-mediated transformation method has achieved transformation of over 50 elite soybean varieties [71]. Third, the use of ternary helper plasmids carrying extra copies of virulence genes or engineered *Agrobacterium* strains capable of delivering imm unosuppressive effector proteins has expanded the host range of recalcitrant species and increased transformation efficiency [72]. However, none of these strategies has been tested in *L. corniculatus* to date, and whether they can substantially broaden the transformable germplasm pool in this species remains an open question requiring direct experimental validation.

Developing precision regulatory tools is essential for achieving spatiotemporal-specific expression. By characterizing *cis*-acting elements within the *L. corniculatus* genome, a comprehensive promoter library—encompassing tissue-specific (roots, leaves, and seeds) and stress-inducible (salinity, drought, and thermal stress) motifs—can be established. Furthermore, marker-free transformation and homologous recombination-mediated site-specific integration techniques need to be developed to avoid gene silencing and growth penalties caused by random insertion, thereby realizing safe, efficient and controllable expression of exogenous genes.

Refining the gene editing system to advance precise reverse genetics represents another key priority. Gene editing enables precise genomic modifications such as insertion, knockout, and base substitution to generate new phenotypes. As a convenient tool, the CRISPR/Cas9-based precise editing system has been widely applied in microorganisms, plants, and animals. To our knowledge, no peer-reviewed reports of successful gene editing in *L. corniculatus* have been published. To address the challenge of editing four alleles simultaneously in tetraploids, CRISPR vectors should be optimized (by incorporating high-fidelity Cas variants and characterizing endogenous promoters) and efficient screening workflows established. Base editing (CBE/ABE) and prime editing technologies hold great potential for achieving precise, single nucleotide modifications, particularly useful for modulating gene function without complete gene knockout. Transient expression of CRISPR reagents—such as ribonucleoprotein delivery or DNA-free methods—enables marker-free editing, eliminating the need for selectable marker genes and addressing biosafety concerns associated with foreign gene integration. However, the lack of an efficient protoplast regeneration system in *L. corniculatus* currently limits the feasibility of DNA-free editing approaches, necessitating the development of delivery systems based on protoplasts or meristematic tissues. Collectively, these advances will provide a standardized and versatile toolkit for high-throughput functional gene characterization and targeted trait improvement in *L. corniculatus*.

Advancing multigene pyramiding improvement and omics-driven breeding is essential for systematic crop enhancement. The development of modular, multi-gene expression vector systems facilitates the synchronized optimization of core agronomic traits, including abiotic stress tolerance, nutritional quality, and symbiotic nitrogen fixation efficiency. Leveraging genome-wide association studies (GWAS) and quantitative trait locus (QTL) mapping, researchers can delineate the underlying regulatory networks governing these complex traits. Integrating these genomic insights with transgenic validation allows for a comprehensive dissection of the molecular mechanisms underlying complex phenotypes, ultimately providing a theoretical framework for molecular assisted gene pyramiding and precision breeding strategies.

Deepening the applications of functional genomics and plant-based bioreactors is an urgent and promising priority for future progress. By leveraging standardized hairy root induction and stable transformation platforms, the functional validation of genes in *L. corniculatus* and heterologous species can be substantially accelerated, while simultaneously advancing fundamental research into symbiotic nitrogen fixation and the molecular mechanisms underlying stress tolerance. In parallel, continued optimization of expression efficiency and biosafety in plant bioreactor systems—encompassing edible vaccines, therapeutic proteins, and related bioproducts—will facilitate the development of low-cost, high-value-added biopharmaceuticals, ultimately broadening the industrial value chain of *L. corniculatus* [10].

In summary, research on genetic transformation of *L. corniculatus*, a leguminous forage species of substantial agronomic and ecological value, has advanced from initial feasibility assessment to a stage centered on improving transformation efficiency, expanding genotype compatibility, and integrating emerging genome-editing tools. Over the coming decade, driven by deep interdisciplinary integration of gene editing, synthetic biology and phenomics, *L. corniculatus* is expected to achieve progress across three directions: (1) refining regeneration protocols and optimizing genotype–vector–strain combinations to broaden applicability across diverse germplasm; (2) accelerating research on plant-based bioreactors to realize the feasibility and stability of producing edible vaccines; (3) leveraging recently obtained genomic resources to accelerate functional validation of candidate genes associated with stress tolerance, forage quality, and symbiotic efficiency. Overcoming existing technical bottlenecks relies on both technical upgrades and mechanistic exploration of regeneration, T-DNA integration and metabolic regulation, facilitating the application of *L. corniculatus* as a multifunctional, high-quality forage in modern bioagriculture.

## Figures and Tables

**Table 2:** Application of genetic transformation in *L. corniculatus*.

Trait Category	Gene	Source Species	Cultivar	Promoter	Construct	Generation Evaluated	Molecular Validation	Function (Biological Evidence) or Application After Being Transferred into *L. corniculatus*	Transformation Method	Reference
Stress tolerance improvement	*AVP1*	*A. thaliana*	‘Mirabel’	NA	*AVP1* overexpression construct	T0	PCR	Enhances compartmentalization of Na^+^ in vacuole, improving salt and drought tolerance	*A. tumefaciens*-mediated	[32]
	*GmNHX1*	*G. max*	‘Leo’	Double CaMV 35S	*GmNHX1* overexpression construct (pGNG)	T0	PCR, Southern blot, RT-PCR	Reduce Na^+^ accumulation and enhance tolerance to NaCl	*A. tumefaciens*-mediated	[20]
	*LcERF056*	*L. corniculatus*	‘Leo’	CaMV 35S	*LcERF056* overexpression (pH7WG2D) and RNAi (pK7GW1WG2) constructs	T0	PCR, qPCR	LcERF056 enhances the salt tolerance by modulating ROS-related genes	*A. tumefaciens*-mediated	[3]
	*ZxNHX* and *ZxVP1-1*	*Z. xanthoxylum*	‘Mirabel’	CaMV 35S	*ZxNHX* and *ZxVP1-1* co-expression construct (pCAMBIA1302); *ZxVP1-1* overexpression construct (pCAMBIA1302)	T0	PCR, RT-PCR	Synergistic improvement in drought and salt tolerance via vacuolar ion compartmentalization	*A. tumefaciens*-mediated	[19]
	*BvSTI*	*B. vulgaris*	‘Bokor’	CaMV 35S	*BvSTI* overexpression construct (pBvSTI)	T0	PCR, DNA gel-blot, RT-PCR, in-gel protein activity assay	Improved salt tolerance, increased tiller number and root weight under salt stress	*A. rhizogenes*-mediated	[53]
	*PeDREB2a*	*P. euphratica*	‘Leo’	rd29A	*KcERF* co-expression construct with *PeDREB2a* (pCAMBIA2301)	T0	PCR	Co-expression significantly enhances tolerance to both drought and salt stress	*A. tumefaciens*-mediated	[17]
	*KcERF*	*K. candel*	‘Leo’	CaMV 35S						
	*TPS1*	*S. cerevisiae*	NA	G10-90	*TPS1* overexpression construct (pX6-TPS1)	T0	PCR	Enhanced leaf water retention, improved drought tolerance in	*A. tumefaciens*-mediated	[54]
	*LcHsp70-1*	*L. corniculatus*	‘Leo’	CaMV 35S	*LcHsp70-1* overexpression construct (pSH737)	T0	GUS staining, PCR, qRT-PCR	Improves heat and cold resistance through protein quality control, antioxidant enhancement, and stress transcription factor modulation	*A. tumefaciens*-mediated	[13]
Quality improvement	*FaMYB1*	*Fragaria* sp.	‘Leo’	Double CaMV 35S	*FaMYB1* overexpression construct	T0, T1	Southern blot, RT-PCR, qRT-PCR	Reduces leaf proanthocyanidins content and optimizes palatability	*A. rhizogenes*-mediated	[5]
	*Sn*	*Z. mays*	‘Leo’	CaMV 35S	*Sn* overexpression construct (121.Sn)	T0	Southern blot, Northern blot, RT-PCR	Significantly increased accumulation of condensed tannins in leaves	*A. rhizogenes*-mediated	[22]
	*DFR*	*A. majus*	‘Leo’	CaMV 35S	*DFR* antisense construct (pMAJ2)	Hairy roots	Southern blot, Slot blot, Northern blot	The antisense DFR reduced tannin levels of “hairy root” cultures	*A. rhizogenes*-mediated	[36]
	*MsGCHI* and *MsADCS*	*M. sativa*	‘Leo’	CaMV 35S (MsGCHI); Gmubi (MsADCS)	*MsGCHI* and *MsADCS* co-expression construct (pCAMBIA1302)	T0	PCR, RT-PCR, RT-qPCR	Co-expression significantly increases folate content and promoting plant growth	*A. tumefaciens*-mediated	[12]
Bioreactor	*P12A-3C*	Foot-and-mouth disease virus (FMDV)	‘Leo’	CaMV 35S	*P12A-3C* overexpression construct (pBin438)	T0	PCR, RT-PCR, ELISA	Successfully integrated and expressed, providing a basis for plant bioreactor production of FMD vaccine	*A. tumefaciens*-mediated	[26]
	*HA*	Avian influenza virus (H5N1)	‘Leo’	CaMV 35S	*HA* overexpression constructs (pBI-MARs-HA, pBI-HA)	T0	PCR, Southern blot, RT-PCR	Successfully integrated and expressed, protein expression confirmed	*A. tumefaciens*-mediated	[6]
	*VP60*	Rabbit hemorrhagic disease virus (RHDV)	NA	CaMV 35S	*VP60* overexpression construct (pBI121)	T0	NA	Regeneration and transformation system established	*A. tumefaciens*-mediated	[24]
	*HBsAg*	Hepatitis B virus	NA	CaMV 35S	*HBsAg* overexpression construct (pBHB)	T0	PCR, Southern blot	The target gene was integrated	*A. tumefaciens*-mediated	[27]
Growth and development regulation	*LcERF1*	*L. corniculatus*	‘Leo’	CaMV 35S	*LcERF1* overexpression construct (pSH737)	T0	GUS staining, PCR, qRT-PCR	Increases endogenous auxin levels, promotes adventitious root formation, number, and elongation.	Transient expression; *A. tumefaciens*-mediated stable transformation	[25]
Agronomic management trait	*bar*	*S. hygroscopicus*	NA	CaMV 35S	*bar* overexpression construct (pCAMBIA3301)	T0	PCR	Confers resistance to glufosinate herbicide	*A. tumefaciens*-mediated	[31]
Symbiotic nitrogen fixation	*lbc3*-*cat*	*G. max*	NA	*lbc3* (2 kb)	*lbc3*-*cat* chimeric reporter construct	T0	Southern blot, Northern blot, primer extension	First nodule-specific foreign gene expression; confirms conserved lb regulation; enables symbiotic engineering	*A. rhizogenes*-mediated	[55]
	*glb3*- *uidA*	*S. rostrata*	NA	*glb3 5′-upstream region*	*glb3*-*uidA* reporter construct (pLP17)	T0	GUS staining, histochemical staining	First functional analysis of *S. rostrata lb* promoter; reveals conserved nodule-specific regulation across legumes	*A. rhizogenes*-mediated	[56]
	*Le1*	*G. max*	NA	*Le1* native promoter/CaMV 35S	*Le1* overexpression constructs (pBIlec, pKYIec)	T0	Immunoblot, immunofluorescence, affinity chromatography	First evidence lectin changes host specificity; confirms role in symbiont recognition	*A. rhizogenes*-mediated	[57]

“NA” indicates the corresponding data were not reported in the original literature.

## Data Availability

No new data were created or analyzed in this study. Data sharing is not applicable to this article.

## Abbreviations

The following abbreviations are used in this manuscript:

SR	Super-growing roots
T-DNA	Transfer DNA
OD	Optical density
Vir	Virulence
AS	Acetosyringone
MS	Murashige and Skoog medium
2,4-D	2,4-Dichlorophenoxyacetic acid
NAA	α-Naphthaleneacetic acid
6-BA	6-Benzylaminopurine
KT	Kinetin
BAP	6-Benzylaminopurine (same as 6-BA)
IBA	Indole-3-butyric acid
5-M-THF	5-methyltetrahydrofolate
nptII	Neomycin phosphotransferase II
PMI	Phosphomannose isomerase
GUS	β-glucuronidase
DsRed2	Discosoma red fluorescent protein 2
ROS	Reactive oxygen species
HSPs	Heat shock proteins
CTs	Condensed tannins
5-F-THF	5-formyltetrahydrofolate
PAs	Proanthocyanidins
CNglcs	Cyanogenic glucosides
MBW	MYB-bHLH-WD40
LAR1	Leucoanthocyanidin reductase 1
DFR	Dihydroflavonol 4-reductase
WT	Wild-type
FMDV	Foot-and-Mouth Disease Virus
HA	Hemagglutinin
RHDV	Rabbit Hemorrhagic Disease Virus
WUS	WUSCHEL
BBM	BABY BOOM
GWAS	Genome-Wide Association Studies
QTL	Quantitative Trait Locus
